# Pancreatic-cancer-cell-derived trefoil factor 2 impairs maturation and migration of human monocyte-derived dendritic cells *in vitro*

**DOI:** 10.1080/19768354.2018.1527721

**Published:** 2018-10-01

**Authors:** Gi-Ho Sung, Hyun Chang, Ji-Yong Lee, Si Young Song, Han-Soo Kim

**Affiliations:** a Institute for Healthcare and Life Science and Institute for Translational and Clinical Research, Catholic Kwandong University International St. Mary’s Hospital, Incheon, Republic of Korea; b Department of Microbiology, Catholic Kwandong University College of Medicine, Gangneung-si, Gangwon-do, Republic of Korea; c Hematology and Medical Oncology, International St Mary’s Hospital, Catholic Kwandong University College of Medicine, Incheon, Republic of Korea; d Department of Anatomy, Yonsei University Wonju College of Medicine, Wonju-si, Gangwon-do, Republic of Korea; e Institute of Gastroenterology, Yonsei University College of Medicine, Seoul, Republic of Korea; f Department of Internal Medicine, Yonsei University College of Medicine, Seoul, Republic of Korea; g Department of Biomedical Sciences, College of Medical Convergence, Catholic Kwandong University, Gangneung-si, Gangwon-do, Republic of Korea

**Keywords:** Chemotaxis, dendritic cells, immunosuppression, oligonucleotide microarray, pancreatic cancer

## Abstract

Pancreatic cancer is a challenging disease with a high mortality rate. While the importance of crosstalk between cancer and immune cells has been well documented, the understanding of this complex molecular network is incomplete. Thus, identification of the secreted proteins contributing to the immunosuppressive microenvironment in pancreatic cancer is crucial for effective diagnosis and/or therapy. We utilized a public microarray dataset (GSE16515) from the Gene Expression Omnibus database to identify genes for secreted proteins in pancreatic cancer. RT–PCR and ELISA of the pancreatic cancer cell lines validated the cellular origin of the selected genes. For functional assay of the selected proteins, we utilized human-monocyte-derived dendritic cells (DCs). From the list of the secreted proteins, trefoil factor 2 (TFF2) was further examined as a potential chemokine/cytokine. While TFF2 did not significantly affect the phenotypic maturation and the allostimulatory capacity of DCs, TFF2 preferentially attracted immature (but not mature) DCs and inhibited their endocytic activity. Our data suggest that TFF2 from pancreatic cancer cells may attract immature DCs and affect the initial stage of DC maturation, thereby contributing to the induction of immune tolerance against pancreatic cancer.

## Introduction

Pancreatic cancer is the fifth leading cause of death from cancer in developed countries. With a poor survival rate of approximately 5%, pancreatic cancer has one of the poorest prognoses among all cancers (Warshaw and Fernández-del Castillo 1992; Magee et al. 2002). Pancreatic ductal adenocarcinoma, the most common form of pancreatic cancer, represents the most lethal type of cancers, with a median survival of 4∼6 months (Saif 2011). This poor survival rate is in part related to pancreatic cancer being generally diagnosed at an advanced stage where effective therapies are lacking. Aside from its silent nature and tendency for late discovery, pancreatic cancer also shows unusual resistance to chemotherapy and radiation therapy. Only 20% of pancreatic cancer patients are eligible for surgical resection, which currently remains the only potentially curative therapy. The lack of efficient molecular markers that can characterize tumor progression precludes making an effective diagnosis, monitoring prognosis, and identifying the therapeutic target of cancers (Lee et al. 2016).

While the immunosuppressive properties of the tumor microenvironment in a number of solid tumors, including pancreatic cancer, have been documented (Dougan 2017), the precise nature and molecular basis of immunosuppression are not well defined. Many mechanisms have been found to contribute to the failure of the immune system to control tumor growth (Kerkar and Restifo 2012). Tumor cells often have decreased expression of major histocompatibility complex (MHC) molecules on their surface (Seliger et al. 1998). Tumor cells are known to produce and secrete many factors into the circulatory system, such as TGF-β, IL-10, prostaglandin E_2_ (PGE_2_), nonfunctional Fas receptors (e.g. RCAS1), and VEGF, which serve to inhibit the function of antigen-presenting cells (APCs) and immune effector cells locally and systemically (von Bernstorff et al. 2001). Most tumor cells lack critical costimulatory molecules, such as CD40, CD80, and CD86, which can contribute to activating T cells (Costello et al. 1999). The prevalence of immunosuppressive regulatory T cells and myeloid-derived suppressor cells (MDSC) in the blood and tumor tissues are contributing factors of pancreatic cancer progression that have also been reported (Hiraoka et al. 2006; Bayne et al. 2012). More recently, the co-inhibitory receptor/ligand system or immune checkpoint proteins (PD-L1/PD-1) expressed on tumor cells and immune cells has emerged as a critical player in the immunosuppression exerted by cancer (Loos et al. 2008; Pillarisetty 2014; Song et al. 2014).

While these immunosuppressive factors lead to reduced numbers and impaired functions of immune effector cells (Bang et al. 2006), the inhibition of dendritic cells (DCs) by a tumor can be the first step in immune evasion by cancer (Pinzon-Charry et al. 2005), as these cells play a pivotal role in the induction and maintenance of an effective immune response (Banchereau et al. 2000). Their function and polarizing capacities are decisive for the outcome of T cell-mediated immunity. In the T cell zone of lymph nodes, they function as APCs, which prime naïve antigen-specific T cells and drive their differentiation toward effector helper T cells and cytotoxic T cells (Banchereau and Steinman 1998). Studies have shown that DCs pulsed with tumor-derived peptides, proteins, or mRNAs are able to substantially augment the anti-tumor immune responses (Shindo et al. 2014; Prue et al. 2015). Gene expression profiling of pancreatic cancer tissues may allow us to acknowledge the tumor microenvironment that is composed of a number of immunosuppressive molecules. Some of these molecules modulate immune response to the tumor by reducing the number and function of circulating dendritic cells (Yanagimoto et al. 2005; Bang et al. 2006). Thus, identifying the molecular signatures of immunosuppressive molecules is critical to understanding the molecular mechanisms underlying this disease and for the development of novel therapeutic strategies. In addition, these will guide to the identification of predictive tumor markers and/or therapeutic targets.

To identify genes that could potentially act as immunosuppressive molecules for pancreatic cancer, the expression of cytokines and chemokines in pancreatic cancer was analyzed on a public microarray dataset and validated on RT–PCR of a number of pancreatic cancer cell lines. The data filtering resulted in a final list of two genes that had little information on pancreatic cancer and immunosuppression: trefoil factor 2 (TFF2) and neuromedin U (NMU). Of these, previous studies (Johnson et al. 2004; Ketterer et al. 2009) suggest that NMU plays a role in the immune response and inflammation, but not in immune suppression. On the other hand, studies have shown that TFF2, also known as a highly conserved secretory protein in gastrointestinal tissues also known as human spasmolytic peptide (SP2), is expressed in lymphoid tissues and is known to be a negative regulator of inflammation and immune cell cytokine responses (Farrell et al. 2002; Baus-Loncar et al. 2005; Kurt-Jones et al. 2007; McBerry et al. 2012; Wills-Karp et al. 2012) and tumorigenesis (Lefebvre et al. 1996; Dubeykovskaya et al. 2016). While it has been suggested that trefoil factors play roles in the immune responses, a previous study demonstrated that TFF3 has no direct effect on LPS-induced murine DC maturation (Loos et al. 2008). However, there are currently no published results regarding the effect of TFF2 on DC function and maturation. Since dysfunction of dendritic cells (DC) by tumor is one of the principal mechanisms of immune escape, we investigated whether TFF2 affects *in vitro* DC maturation and function.

## Materials and methods

### Microarray data processing

A public dataset was obtained from the Gene Expression Omnibus database (http://www.ncbi.nlm.nih.gov/geo/) (Barrett and Edgar 2006). Specifically, dataset GSE16515 ([HG-U133_Plus_2] Affymetrix Human Genome U133 Plus 2.0 Array) (Pei et al. 2009) consisted of 36 pancreatic cancer tissue samples and 16 matched normal pancreatic tissue samples. Normalization between samples was performed using the preprocess Affy package of R/Bioconductor (Gentleman et al. 2004). After data preprocessing, differential expression analysis between pancreatic cancer and normal samples was performed using the multi-test package of R/Bioconductor (Pollard and van der Laan 2005) with a fold change >2 and a *p* value ≤ 0.05 as strict thresholds. A hierarchical heatmap was generated using heatmap.2 from the R package gplots (http://cran.r-project.org/web/packages/gplots/index.html). The selected DEGs list was submitted to the DAVID (Database for Annotation, Visualization and Integrated Discovery) online free tool (http://david.abcc.ncifcrf.gov/home.jsp) to perform functional annotation based on gene ontology (Dennis et al. 2003), and pathway enrichment analysis based on KEGG (Kyoto Encyclopedia of Genes and Genomes).

### Reagents

The culture media used were RPMI-1640, IMDM, McCoy’s 5a, DMEM, or Ham’s F-12. These media were supplemented with 2 mM L-glutamine, 20 mM HEPES, 1% antibiotic-antimycotic solution (all obtained from Invitrogen, Carlsbad, CA, USA), and 10% heat-inactivated fetal bovine serum (FBS) (HyClone, Logan, UT, USA). Recombinant human GM-CSF, IL-4, TFF2, IL-8, and MIP-3β were obtained from Peprotech (Peprotech, Rocky Hill, NJ, USA). LPS was from Sigma Chemical Co. (St. Louis, MO, USA). The following fluorochrome-labeled monoclonal antibodies were used to analyze phenotypes of cells in peripheral blood mononuclear cells (PBMC) or cultured DC: CD1a-PE, CD40-FITC, CD80-PE, CD83-FITC, CD86-PE, and HLA-DR-FITC (all from BD-Pharmingen, San Jose, CA, USA).

### Cell lines

AsPC-1, BxPC-3, Capan-1, Capan-2, CFPAC-1, HPAC, MiaPaCa-2, PANC-1, Panc 03.27, and Panc 02.13 were obtained from the American Type Culture Collection (ATCC, Rockville, MD, USA). SNU-213, SNU-324, and SNU-410 were obtained from Korean Cell Line Bank (Seoul, Korea). SNU-213, SNU-324, BxPC-3, Panc 03.27, Panc 02.13 cells (all primary tumor-derived), AsPC-1 (ascite-derived), and SNU-410 (from liver metastasis) were grown in RPMI1640 with 10% FBS. Capan-1 and CFPAC-1 cells (both from liver-metastasis-derived) were grown in IMDM with 10% FBS. Capan-2 cells (primary tumor-derived) were grown in McCoy’s 5a with 10% FBS. PANC-1 and MIA PaCa-2 cells (primary tumor-derived) were grown in DMEM with 10% FBS and 2.5% horse serum. HPAC cells were maintained in DMEM/F-12 with 5% FBS. This study was approved by the IRB of International St. Mary’s Hospital (Incheon, Korea). All cultures were maintained at 37°C in a humidified atmosphere containing 95% air and 5% CO_2_. For RNA isolation, cells were washed 3 times with PBS and harvested with Trypsin/EDTA (Invitrogen).

### RT–PCR

RNA was extracted using a QIAshredder and the RNeasy kit (Qiagen, Valencia, CA, USA) according to the manufacturer’s instructions. RT–PCR was performed using the following primers specific for TFF2 (forward 5’-AGTGAGAAACCTCCCCC-3’ and reverse 5’-AACACCCGGTGAGCCAC-3’) and β-actin (forward 5’- CATGTACGTTGCTATCCAGGC -3’ and reverse 5’- CTCTCTTAATGTCACGCACGAT -3’). Amplification was performed with 30 cycles at 94°C for 30 s, 57°C for 30 s, and 72°C for 45 s, with a final extension step at 72°C for 10 min. After visualization of PCR products electrophoresed on a 1.5% agarose gel, gel images were obtained using the image analyzer (LAS-1000; Fuji Photo Film Co., Tokyo, Japan).

### Enzyme-linked immunosorbent assay (ELISA)

Secreted TFF2 protein levels in the culture supernatants of the pancreatic cancer cell lines were quantified using Human TFF2 DuoSet (R&D Systems, Minneapolis, MN, USA) according to the manufacturer’s instructions.

### DC generation and maturation

Healthy donors enrolled in the study gave written informed consent prior to the procedure. The study protocol was approved by the Institutional Review Board of Severance Hospital and met the guidelines for blood donation. Peripheral blood mononuclear cells (PBMCs) from healthy donors were prepared by density centrifugation on a Ficoll-Paque gradient (Pharmacia Biotech, Uppsala, Sweden). Monocytes were purified from PBMC by positive isolation using anti-CD14 conjugated magnetic microbeads (MACS CD14 isolation kit, Miltenyi Biotech, Bergisch Gladbach, Germany). Purity was checked by flow cytometer with anti-CD45-FITC and anti-CD14-PE antibodies, and was routinely > 95%.

Human monocyte-derived DCs were generated with GM-CSF (100 ng/ml) and IL-4 (20 ng/ml) for 6 days at 37°C in a 5% CO_2_ atmosphere. Cultures were fed on Day 3 by adding fresh medium with cytokines. On Day 6, immature DCs (iDCs) were stimulated with LPS (500 ng/ml) to mature DCs (LPS-DC). In some experiments, TFF2 or IL-10 was added to investigate their inhibitory effect on DC maturation or function.

### Flow cytometric analysis

For flow cytometry, DCs were stained with various monoclonal antibodies or isotype control antibodies for 15 min at 4°C in the dark. The cells were washed in PBS and then fixed in PBS containing 1% paraformaldehyde. For data analysis, a Cytomics^TM^ Flow Cytometer (Beckman Coulter, Fullerton, CA, USA) was used. The DC population was gated based on its forward-scatter and side-scatter profile and the data were analyzed with the Flowing Software (version 2.5.1, Turku Centre for Biotechnology, Turku, Finland, http://flowingsoftware.btk.fi/).

### In vitro DC functional assays

The immunostimulatory capacity of DCs was assessed by allogenic mixed leukocyte reaction (MLR). A total of 2 × 10^5^ allogeneic T cells were incubated with irradiated DCs (30 Gy) at different responder:stimulator ratios ranging between 10:1 and 1280:1 in 96-well flat-bottom plates. After 4 days of coculture, [^3^H] thymidine (0.5 μCi/well) was added during the last 16 h. The amount of [^3^H] thymidine incorporation was measured by liquid scintillation (Wallac, Waltham, MA, USA). Responses were reported as the mean of triplicate counts per minute (cpm) ± SD, less the background counts.

Receptor-mediated endocytosis of DCs was assessed using FITC-tagged dextran. Immature DCs were harvested on Day 5 and incubated with FITC-dextran (20 μg/ml), either at 4°C (internalization control) or at 37°C, for 30 min. The cells were then acquired using the flow cytometer.

For chemotaxis, migration of DCs in response to chemotactic factors was assessed using 24-well transwell plates with polycarbonate filters of 5 μm pore size (Corning Costar, New York, NY, USA). Cells were washed 3 times and resuspended in RPMI 1640. TFF2 (final concentration of 10 ng/ml∼10 μg/ml) in 600 µl of serum-free RPMI-1640 was placed in the lower compartment of the chambers, and 200 µl of cell suspension (5 × 10^5^ cells) was added to the upper compartment. DC migration towards MIP-3β (10 ng/ml) or IL-8 (50 ng/ml) was used as a control for mDC and iDC, respectively. Cells were allowed to migrate at 37°C for 2 h, after which time the migrated cells in bottom chamber were collected and counted by hemocytometer. Alternatively, the transmigrated cells were collected from the lower chamber, fixed, and counted on a flow cytometer.

### Statistical analysis

Statistical significance was determined by either a unpaired Student’s *t*-test, one-way or two-way ANOVA with a Bonferroni’s post-test using GraphPad Prism software, version 5.01 (GraphPad Software, Inc., La Jolla, CA, USA). *P*-values < 0.05 were considered statistically significant.

## Results

### Identification of candidate genes from microarray dataset

**Figure 1.**
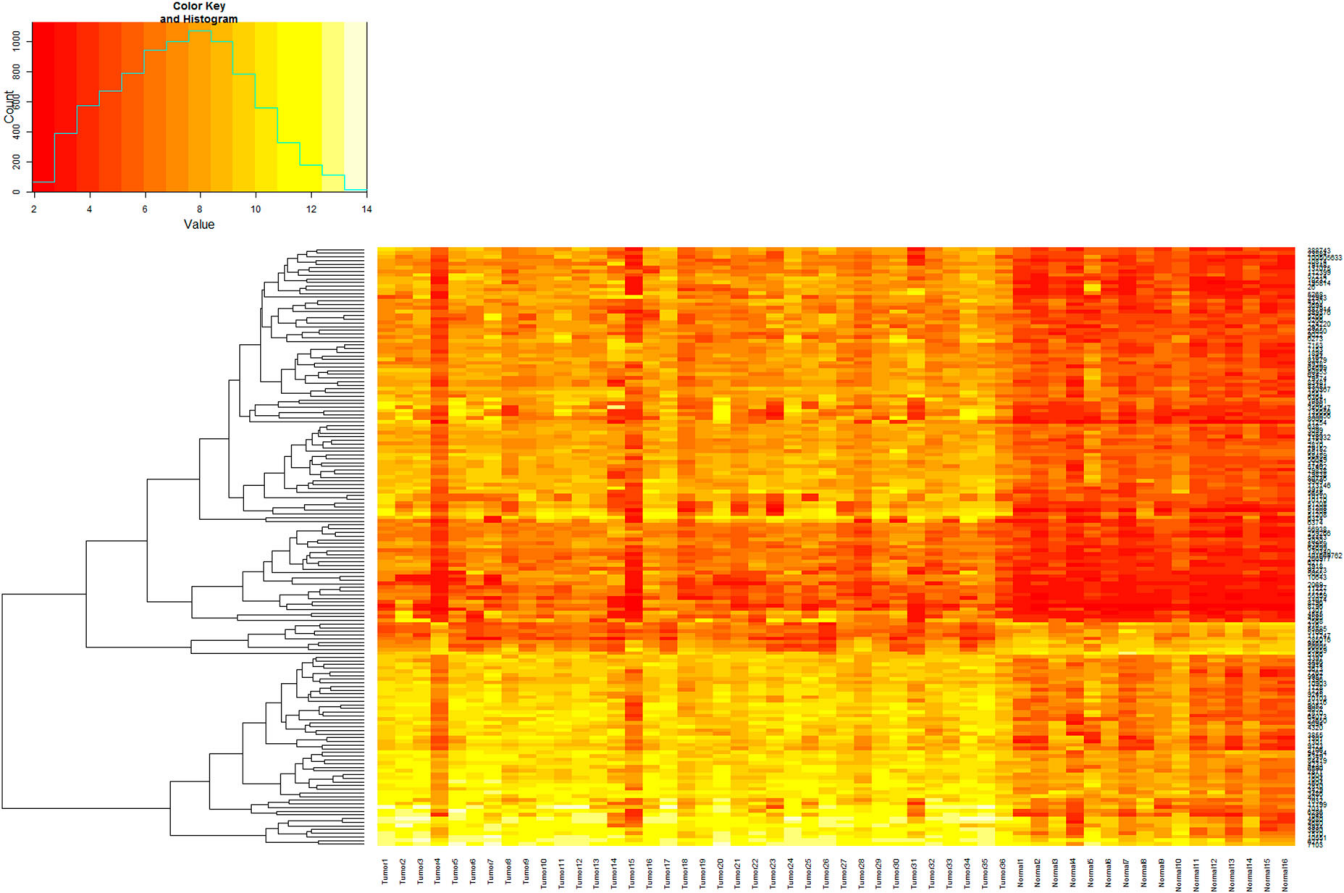
Unsupervised hierarchical clustering of 52 samples based on expression levels detected in the microarray experiment using the 163 probes differentially expressed between pancreatic cancer (PC) and control samples. PC samples are in blue and control samples are in black. Samples are clustered on the horizontal axis and genes (probes) are clustered on the vertical axis. The lengths of the branches in the dendrograms represent the degrees of correlation between samples or gene sets. For expression levels, yellow represents overexpressed genes and red represents underexpressed genes.

We examined the gene expression profiles of pancreatic cancer and normal pancreatic tissues of a public dataset (GSE16515). To identify differentially expressed genes, we performed a fold-change filtering between the cancer and normal samples. We first selected outlying genes that have an average expression ratio >2.0 SD from the mean. This 2.0 SD cutoff represents a ≥ 95% confidence interval. Since the overexpressed genes represent greater potential as targets for drug design and diagnostic perspectives, we focused on the validation of overexpressed genes. A total of 163 probes representing 127 genes were identified as being significantly upregulated in cancer patient samples compared to those of the normal controls (Table 1). Unsupervised hierarchical clustering using these probes showed good delineation between pancreatic cancer patients and normal controls (Figure 1).

**Table 1.** Upregulated genes in pancreatic cancer tissues.

ID	Symbol	ID	Symbol	ID	Symbol
1555731_a_at	AP1S3	205927_s_at	CTSE	220030_at	STYK1
1555950_a_at	CD55	205960_at	PDK4	220177_s_at	TMPRSS3
201250_s_at	SLC2A1	206023_at	NMU	220658_s_at	ARNTL2
201291_s_at	TOP2A	206482_at	PTK6	221132_at	CLDN18
201292_at	TOP2A	206884_s_at	SCEL	221133_s_at	CLDN18
201467_s_at	NQO1	207517_at	LAMC2	222608_s_at	ANLN
201468_s_at	NQO1	207850_at	CXCL3	223278_at	GJB2
201650_at	KRT19	208083_s_at	ITGB6	223484_at	C15orf48
201884_at	CEACAM5	208170_s_at	TRIM31	223631_s_at	C19orf33
201925_s_at	CD55	208937_s_at	ID1	223748_at	SLC4A11
201926_s_at	CD55	209016_s_at	KRT7	223949_at	TMPRSS3
202267_at	LAMC2	209114_at	TSPAN1	223952_x_at	DHRS9
202411_at	IFI27	209173_at	AGR2	224009_x_at	DHRS9
202489_s_at	FXYD3	209260_at	SFN	224428_s_at	CDCA7
202504_at	TRIM29	209270_at	LAMB3	225207_at	PDK4
202831_at	GPX2	209498_at	CEACAM1	225436_at	ABHD17C
202856_s_at	SLC16A3	209792_s_at	KLK10	226535_at	ITGB6
202934_at	HK2	209803_s_at	PHLDA2	227314_at	ITGA2
203021_at	SLPI	209950_s_at	VILL	227475_at	FOXQ1
203108_at	GPRC5A	210095_s_at	IGFBP3	228058_at	ZG16B
203510_at	MET	210143_at	ANXA10	228232_s_at	VSIG2
203559_s_at	AOC1	210519_s_at	NQO1	228707_at	CLDN23
203691_at	PI3	211002_s_at	TRIM29	228846_at	MXD1
203726_s_at	LAMA3	211657_at	CEACAM6	228923_at	S100A6
203757_s_at	CEACAM6	212143_s_at	IGFBP3	228969_at	AGR2
203819_s_at	NA	212236_x_at	NA	229030_at	CAPN8
203820_s_at	IGF2BP3	212444_at	GPRC5A	229271_x_at	COL11A1
203824_at	TSPAN8	212531_at	LCN2	229490_s_at	NA
203876_s_at	MMP11	212657_s_at	IL1RN	229927_at	LEMD1
203878_s_at	MMP11	212942_s_at	CEMIP	230493_at	SHISA2
204170_s_at	CKS2	212992_at	AHNAK2	231646_at	DPCR1
204268_at	S100A2	214135_at	CLDN18	231944_at	ERO1LB
204320_at	COL11A1	214385_s_at	MUC5AC	232056_at	SCEL
204351_at	S100P	214476_at	TFF2	232105_at	BLACAT1
204424_s_at	LMO3	214974_x_at	CXCL5	232164_s_at	EPPK1
204602_at	DKK1	215034_s_at	TM4SF1	232165_at	EPPK1
204614_at	SERPINB2	215101_s_at	CXCL5	232578_at	CLDN18
204653_at	TFAP2A	215125_s_at	NA	236129_at	GALNT5
204855_at	SERPINB5	217109_at	MUC4	237183_at	GALNT5
204885_s_at	MSLN	217110_s_at	MUC4	238017_at	SDR16C5
205009_at	TFF1	217728_at	S100A6	238018_at	FAM150B
205044_at	GABRP	218332_at	BEX1	238439_at	ANKRD22
205076_s_at	MTMR11	218677_at	S100A14	238689_at	GPR110
205081_at	CRIP1	218960_at	TMPRSS4	239272_at	MMP28
205083_at	AOX1	219014_at	PLAC8	239370_at	LINC01133
205157_s_at	NA	219232_s_at	EGLN3	240303_at	TMC5
205319_at	PSCA	219404_at	EPS8L3	241137_at	DPCR1
205466_s_at	HS3ST1	219429_at	FA2H	243764_at	VSIG1
205476_at	CCL20	219508_at	GCNT3	244056_at	SFTA2
205552_s_at	OAS1	219529_at	CLIC3	244780_at	SGPP2
205597_at	SLC44A4	219787_s_at	ECT2	33322_i_at	SFN
205767_at	EREG	219795_at	SLC6A14	33323_r_at	SFN
205771_s_at	AKAP7	219915_s_at	SLC16A10	37892_at	COL11A1
205780_at	BIK	219918_s_at	ASPM	41469_at	PI3

As shown in Table 2, upregulated genes were mostly enriched in 5 BP (biological processes) terms: ectoderm development (GO:0007398), epidermis development (GO:0008544), digestion (GO:0007856), cell adhesion (GO:0007155), and response to external stimulus (GO:0009605). For the CC (cellular components) term, upregulated genes were significantly enriched in the extracellular region (GO:0005576), extracellular region part (GO:0055532), proteinaceous extracellular matrix (GO:0005578), plasma membrane part (GO:00044459), and integral to plasma membrane (GO:0005887). For the MF (molecular functions) term, structural molecular activity (GO:0005198), endopeptidase activity (GO:0004175), serine-type endopeptidase inhibitor activity (GO:0004867), receptor binding (GO:0005102), and calcium ion binding (GO:0005509) were most significant for upregulated genes.

**Table 2.** GO pathway enrichment analysis of upregulated genes in pancreatic cancer cells^a^.

GO	Term	Count	*P* value	Genes
BP	GO:0007398 ectoderm development	10	9.00E-06	FOXQ1, LAMC2, EREG, AHNAK2, LAMA3, TFAP2A, LAMB3, SFN, SCEL, Unknown
CC	GO:0005576 extracellular region	32	3.79E-05	LAMC2, EREG, CCL20, CXCL3, TFF1, AOC1, SFTA2, MMP28, PI3, LAMB3, KLK10, ZG16B, MUC5AC, IL1RN, CXCL5, SFN, COL11A1, FAM150B, MMP11, MUC4, NMU, DKK1, SLPI, LAMA3, LCN2, IGFBP3, AGR2, MSLN, CRECAM1, MUC4, SERPINB2, TFF2
BP	GO:0008544 epidermis development	9	3.85E-05	FOXQ1, LAMC2, EREG, AHNAK2, LAMA3, LAMB3, SFN, SCEL, Unknown
CC	GO:0044421 extracellular region part	20	7.06E-05	LAMC2, EREG, CCL20, CXCL3, TFF1, AOC1, LAMC2, MMP28, PI3, LAMB3, MUC5AC, IL1RN, CXCL5, SFN, COL11A1, MMP11, MUC4, LAMA3, IGFBP3, SERPINB2
BP	GO:0007586 digestion	6	4.03E-04	MUC5AC, Unknown, NMU, CTSE, TFF1, TFF2
BP	GO:0030216 keratinocyte differentiation	5	0.00109	SFN, EREG, SCEL, AHNAK2, LAMA3
BP	GO:0009913 epidermal cell differentiation	5	0.00151	SFN, EREG, SCEL, AHNAK2, LAMA3
MF	GO:0005198 structural molecule activity	12	0.00241	EPPK1, CLDN23, MUC4, KRT19, COL11A1, KRT7, LAMA3, CLDN18, VILL, LAMB3, MUC5AC, Unknown
CC	GO:0005578 proteinaceous extracellular matrix	9	0.00251	MUC4, LAMC2, PI3, COL11A1, LAMA3, MMP28, MMP11, LAMB3, MUC5AC
BP	GO:0030855 epithelial cell differentiation	6	0.00253	SFN, DHRS9, EREG, SCEL, AHNAK2, LAMA3
BP	GO:0007155 cell adhesion	13	0.003	LAMC2, CLDN23, MUC4, KRT19, ITGB6, COL11A1, LAMA3, CLDN18, CLDN18, CLDN18, ITGB6, LAMC2, LAMB3, MUC5AC, ITGA2, COL11A1, MSLN, CRECAM1, MUC4, CLDN18, COL11A1
BP	GO:0022610 biological adhesion	13	0.00304	LAMC2, CLDN23, MUC4, KRT19, ITGB6, COL11A1, LAMA3, CLDN18, LAMB3, MUC5AC, ITGA2, MSLN, CRECAM1
CC	GO:0016323 basolateral plasma membrane	7	0.00388	MET, ITGA2, SLC4A11, SLC2A1, CEACAM5, LAMA3, SLC16A10
CC	GO:0031012 extracellular matrix	9	0.00397	MUC4, LAMC2, PI3, COL11A1, LAMA3, MMP28, MMP11, LAMB3, MUC5AC
CC	GO:0016328 lateral plasma membrane	3	0.00461	KRT19, AKAP7, GJB2
CC	GO:0044459 plasma membrane part	28	0.00463	Unknown, CLDN23, LAMC2, KRT19, ITGB6, EREG, CEACAM5, AKAP7, CEACAM6, CLDN18, ITGA2, SLC4A11, VSIG2, SLC6A14, CD55, GPRC5A, TM4SF1, SLC16A3, MUC4, SLC2A1, LAMA3, FXYD3, AP1S3, GABRP, MET, CRECAM1, GJB2, SLC16A10
BP	GO:0060429 epithelium development	7	0.00474	SFN, DHRS9, EREG, SCEL, AHNAK2, LAMA3, TFAP2A,
BP	GO:0009888 tissue development	12	0.00585	FOXQ1, LAMC2, DHRS9, EREG, AHNAK2, COL11A1, LAMA3, TFAP2A, LAMB3, SFN, SCEL, Unknown
BP	GO:0048513 organ development	22	0.0066	FOXQ1, LAMC2, EREG, DHRS9, AHNAK2, ASPM, LAMB3, ITGA2, SFN, SCEL, COL11A1, PHLDA2, Unknown, BIK, DKK1, LAMA3, TFAP2A, MET, CRECAM1, HK2, ID1, GJB2
BP	GO:0043542 endothelial cell migration	3	0.00987	ID1, S100A2, S100P
MF	GO:0004175 endopeptidase activity	8	0.01032	CAPN8, CTSE, TMPRSS3, TMPRSS4, MMP11, MMP28, SERPINB2, KLK10
CC	GO:0044420 extracellular matrix part	5	0.0109	MUC5AC, LAMC2, COL11A1, LAMA3, LAMB3
CC	GO:0005615 extracellular space	12	0.01211	LAMC2, EREG, CXCL3, CCL20, TFF1, AOC1, IGFBP3, IL1RN, CXCL5, SFN, SFN, SERPINB2,
BP	GO:0048856 anatomical structure development	27	0.01881	FOXQ1, LAMC2, DHRS9, EREG, AHNAK2, ASPM, LAMB3, ITGA2, SFN, COL11A1, Unknown, SCEL, PHLDA2, BEX1, BIK, DKK1, LAMA3, S100A6, TFAP2A, IGFBP3, MET, ECT2, IGF2BP3, CRECAM1, ID1, HK2, GJB2
MF	GO:0004867 serine-type endopeptidase inhibitor activity	3	0.02123	PI3, SLPI, SERPINB2,
BP	GO:0009605 response to external stimulus	13	0.02249	Unknown, ITGB6, EREG, ARNTL2, CXCL3, CCL20, COL11A1, ITGA2, IL1RN, CXCL5, AOX1, CD55, SERPINB2
CC	GO:0031225 anchored to membrane	6	0.02327	CEACAM5, AKAP7, MSLN, CEACAM6, PSCA, CD55
BP	GO:0048731 system development	25	0.02403	FOXQ1, LAMC2, EREG, DHRS9, AHNAK2, ASPM, LAMB3, ITGA2, SFN, SCEL, COL11A1, PHLDA2, Unknown, BEX1, BIK, DKK1, S100A6, LAMA3, TFAP2A, IGFBP3, MET, CRECAM1, HK2, ID1, GJB2
MF	GO:0005102 receptor binding	12	0.02605	MUC4, ITGB6, NMU, EREG, DKK1, CXCL3, TFF1, CCL20, LAMA3, ITGA2, IL1RN, CXCL5
CC	GO:0005887 integral to plasma membrane	16	0.02761	Unknown, MUC4, ITGB6, EREG, CEACAM5, CEACAM6, FXYD3, MET, ITGA2, VSIG2, CRECAM1, SLC6A14, TM4SF1, CD55, GPRC5A, SLC16A3

^a^Top 30 terms were chosen according to the *P* value. GO gene ontology.

### Selection of immune-associated genes highly expressed in pancreatic cancer and validation in pancreatic cancer cell lines

Next, we investigated the functional distribution of the 127 genes that were upregulated in pancreatic cancer. We observed a list of 106 transcripts associated with the following GO terms: extracellular region, extracellular matrix, and extrinsic to plasma membrane. These terms were followed with the keywords of ligands, cytokines, growth factors and extracellular matrix (Table 3).

**Table 3.** Classification of genes for secreted proteins that are upregulated in pancreatic cancer cells.

Functional Categories	Gene Symbol
Growth factor/ Ligand	ITGB6, EREG, ARNTL2, CXCL3, CCL20, ITGA2, IL1RN, CXCL5, AOX1, SERPINB2, NMU, DKK1, TFF1,TFF2
Secreted	LAMC2, EREG, CCL20, CXCL3, TFF1, AOC1, SFTA2, MMP28, PI3, LAMB3, KLK10, ZG16B, MUC5AC, IL1RN, CXCL5, SFN, COL11A1, FAM150B, MMP11, MUC4, NMU, DKK1, PI3, SLPI, LAMA3, LCN2, MMP11, IGFBP3, AGR2, MSLN, CRECAM1, AGR2, SERPINB2, TFF2
Extracellular matrix	MUC4, LAMC2, PI3, COL11A1, LAMA3, MMP28, PI3, LAMB3, MUC5AC, MMP11

**Figure 2.**
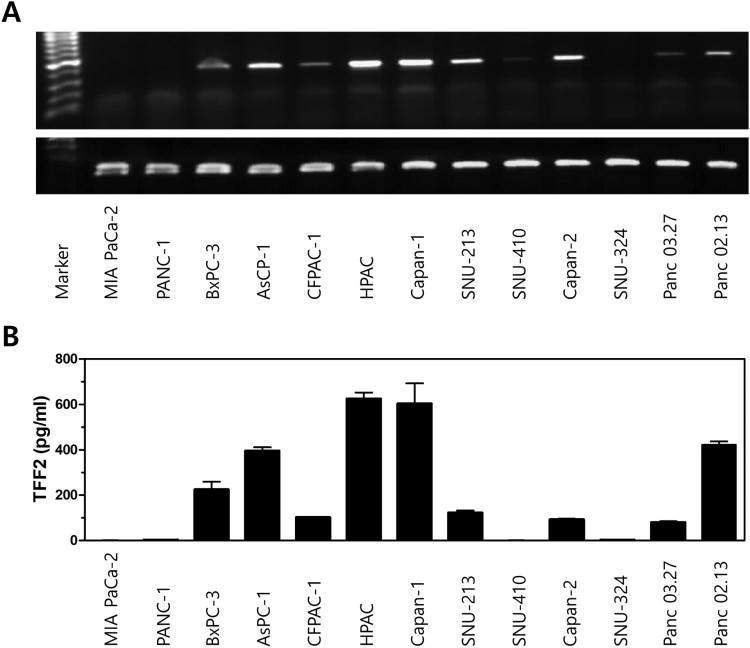
The expression of TFF2 in various pancreatic cancer cell lines. (A) TFF2 transcript was detected in 9 of 13 human pancreatic cancer cell lines by RT-PCR. β-actin was used to control for the amount of amplified cDNA. The results shown are from one representative of three independent experiments performed. (B) ELISA detected TFF2 that had been secreted into the culture medium of human pancreatic cancer cell lines. The results shown are from one representative of three independent experiments.

The gene lists were compared with the public database of gene expression profiles from 21 human pancreatic cancer cell lines [https://www.ebi.ac.uk/arrayexpress/experiments/E-GEOD-40099/] and the online NCBI database PubMed for reference search. The majority of the 106 genes were previously reported as pancreas- or pancreatic-cancer-associated genes. Many of them were typically associated with general metabolism of the pancreas, pancreatitis, and pancreatic cancer. The data filtering narrowed the number of genes to a final list of four that had limited information on pancreatic cancer as well as immunosuppression: secreted phosphoprotein 1 (SPP1, osteopontin), granulin, trefoil factor 2 (TFF2), and neuromedin U (NMU). Of these proteins, we selected TFF2 as it is a relatively unexplored gene for its immunosuppressive function, and we also examined the role of TFF2 in DC maturation and function. Comparison of TFF2 gene expression in normal pancreas and pancreatic tumor samples revealed a 2.9-fold increase in tumors compared to that of normal pancreatic tissues. In order to validate the cellular origin of TFF2, we performed RT–PCR of the selected genes with human pancreatic cancer cell lines. As shown in Figure 2(A), the expression of TFF2 was verified in 9 of 13 cell lines tested. TFF2 expression was detected in BxPC-3, AsPc-1, Capan-1, CFPAC, HPac, Capan-1, SNU-213, Capan-2, Panc 03.27, and Panc 02.13, implying that TFF2 selected from genome-wide expression profiles is mainly expressed by tumor cells in the utilized tissue specimens. The expression and secretion of TFF2 protein from these cells were further validated by TFF2 ELISA (Figure 2(B)). Cells expressing high levels of TFF2 mRNA secreted a comparably high level of TFF2 proteins into the culture medium. However, there was no correlation between TFF2 expression levels and the tumor cell lines obtained from primary and secondary (metastatic or ascite-derived) tissues.

### TFF2 impair differentiation and function of dendritic cells

**Figure 3.**
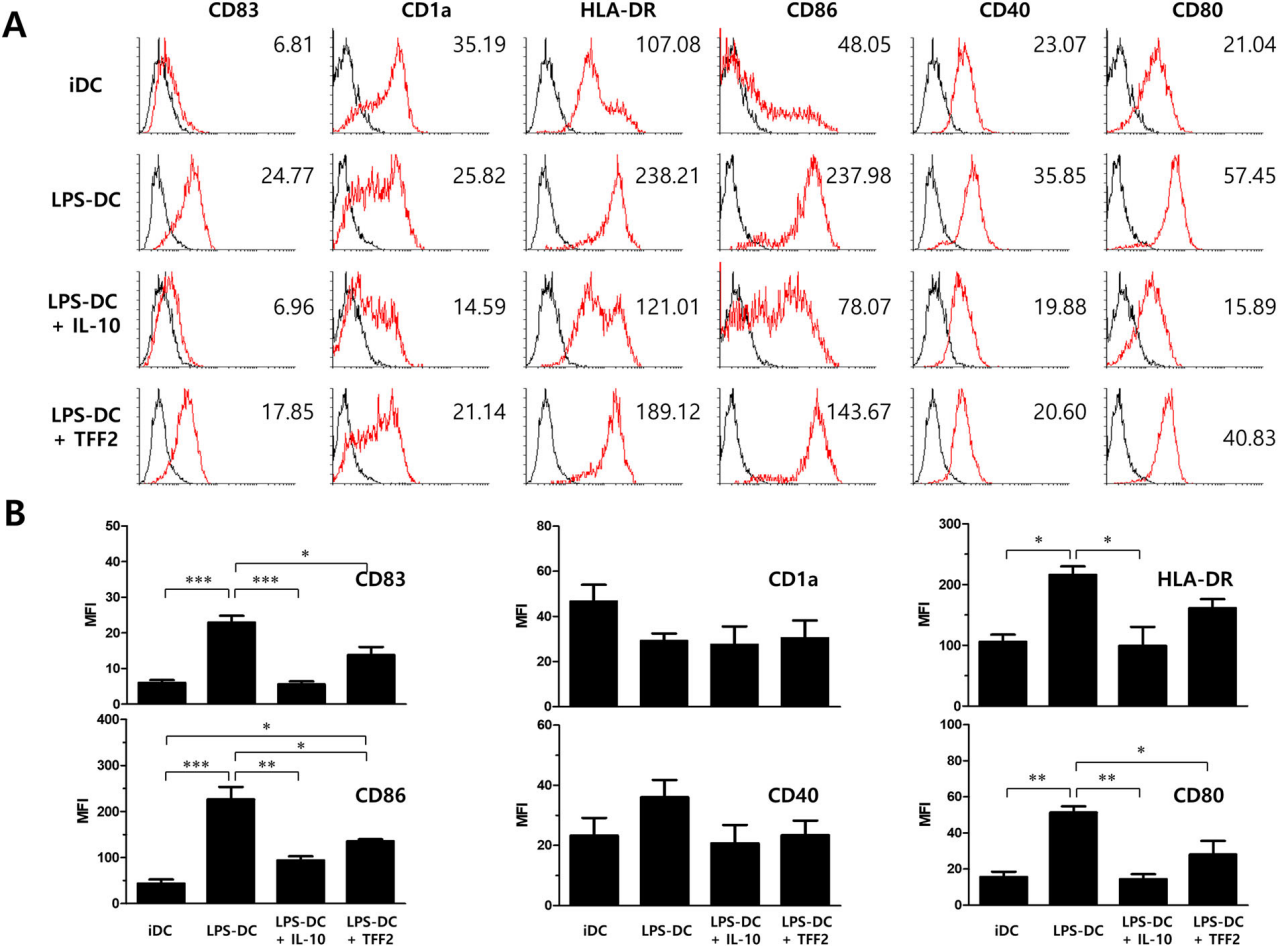
Treatment of monocyte-derived dendritic cells with TFF2 during maturation slightly alters the phenotype of monocyte-derived DCs. (A) Human monocyte-derived (iDCs) were cultured with LPS (1 μg/ml) in the presence or absence of IL-10 (10 ng/ml) or TFF2 (1 μg/ml) for 48 h and stained with antibodies against HLA-DR, CD40, CD1a, CD80, CD83, and CD86 to determine the phenotype of DCs. Histograms represent the overlay image of corresponding antibody staining (red line) and matching isotype control antibody staining (black line) with geometric mean fluorescence intensity (MFI). The results shown are from one representative of three independent experiments performed. (B) Statistical significance of the DC maturation marker expression in the cultured DCs in the presence of LPS, IL-10, or TFF from three independent experiments was determined using one-way ANOVA with a Bonferroni’s post-test. (*p < 0 0.05, **p < 0 0.01 and *** *p *< 0 0.001)

Studies have proposed a role for trefoil factors (TFFs) in regulating immune responses (Cook et al. 1999; Hedrick et al. 2000; Makarenkova et al. 2003; Baus-Loncar et al. 2005; Moriyama et al. 2006; Loos et al. 2008). To investigate whether TFF2 can modulate DC maturation, monocyte-derived iDCs were cultured with GM-CSF, IL-4, and LPS in the presence or absence of TFF2 for 48 h. Because LPS-induced maturation of DCs is inhibited by IL-10 (Steinbrink et al. 1997; McBride et al. 2002), DCs pre-treated with IL-10 were used as a positive control for inhibition of maturation. The phenotype of the different DC groups was determined by flow cytometry. Mature DCs are known to express higher levels of CD83, HLA-DR, CD80, CD86, and CD40 than immature DCs. Non-treated (immature DCs, iDCs) and TFF2-treated DCs expressed similar levels of co-stimulatory molecules in the absence of LPS, while IL-10 treatment led only to reduction of HLA-DR (data not shown). While LPS treatment induced maturation of DCs (LPS-DC) by up-regulation of HLA-DR, CD40, CD83, CD86, and CD80, IL-10 treatment to the LPS-stimulated DC dramatically down-regulated the expression of these markers comparable to the levels expressed by control iDCs. In contrast, TFF2 treatment in the presence of LPS significantly reduced the expression of the key maturation markers CD86, CD80, and CD83 (Figure 3).

**Figure 4.**
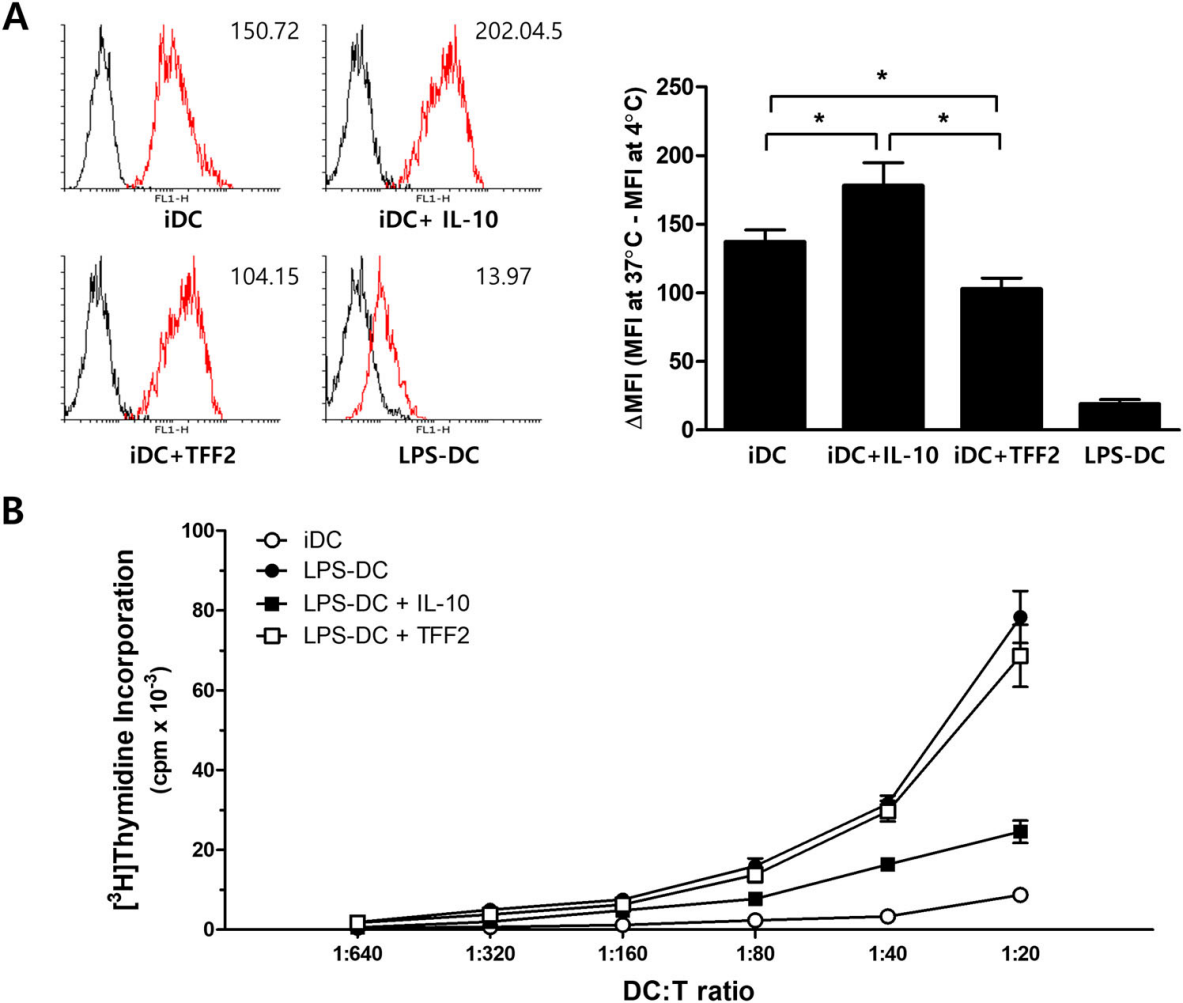
TFF2 affects the function of immature human dendritic cells (iDCs). (A) TFF2 suppresses the endocytic activity of iDCs. Immature DCs were left untreated or treated with IL-10, TFF2, or LPS as described, and FITC-dextran uptake was subsequently measured by flow cytometry. Data are shown as representative histograms of FITC-dextran uptake at both 37°C (red line) and 4°C (black line, control) and as mean ΔMFI (MFI at 37°C – MFI at 4°C) ± SEM for three independent experiments. Values of *p* were calculated using two-way ANOVA, *p < 0 0.05 and ***p < 0 0.001. (B) TFF2 did not affect the allostimulatory function of mature DCs. Human monocyte-derived iDCs were cultured with LPS (1 μg/ml) in the presence or absence of IL-10 (10 ng/ml) or TFF2 (1 μg/ml) for 48 h. mDCs were harvested and used to stimulate allogeneic T cells. T cells were cocultured with DCs at various stimulator:responder ratios for 5 days, and cell proliferation was measured by [^3^H]thymidine uptake for the last 16 h. Results are shown as mean cpm ± SD of triplicate determinations and are representative of three independent experiments performed. *P < 0.05 and **P < 0.01 versus that of LPS-stimulated DC.

The primary role of immature DCs is to capture and process antigens with their phagocytosis/endocytosis capacity that is developmentally regulated during maturation (Steinman et al. 1997). We assessed the receptor-mediated endocytic function of DCs using Dextran-FITC by DCs treated with TFF2, IL-10, and untreated DCs. The antigen uptake was very high in immature DCs (iDCs) (mean fluorescence intensity of 137.0 ± 15.3, *n* = 3); the addition of IL-10 further enhanced the endocytic activity of DCs (177.9 ± 29.3). However, TFF2 treatment significantly reduced the activity (102.7 ± 14.0) (Figure 4).

Allostimulatory capacity is one of the key functional characteristics of mature DCs. To test whether the mature phenotype of the DCs after LPS treatment correlates with their capacity to stimulate T cells, DCs were co-cultured with allogeneic purified CD4^+^ T cells. Consistent with phenotypic profile after LPS stimulation, IL-10-treated DCs in the presence of LPS were less effective in activating allogeneic CD4^+^ T cells than control mDCs (LPS-DC) (Figure 4(B)). In contrast, the allostimulatory activity of TFF2-treated DCs in the presence of LPS was comparable to that of LPS-DCs, suggesting that TFF2 does not affect the T cell stimulatory capacity of DCs. Thus, our data indicate that addition of TFF2 does not substantially alter the DC maturation induced by LPS. Although LPS induction of IL-12p70, an essential cytokine for DC maturation or activation, was significantly reduced in the presence of IL-10, TFF2 did not affect the production of this cytokine in the presence of LPS (data not shown).

**Figure 5.**
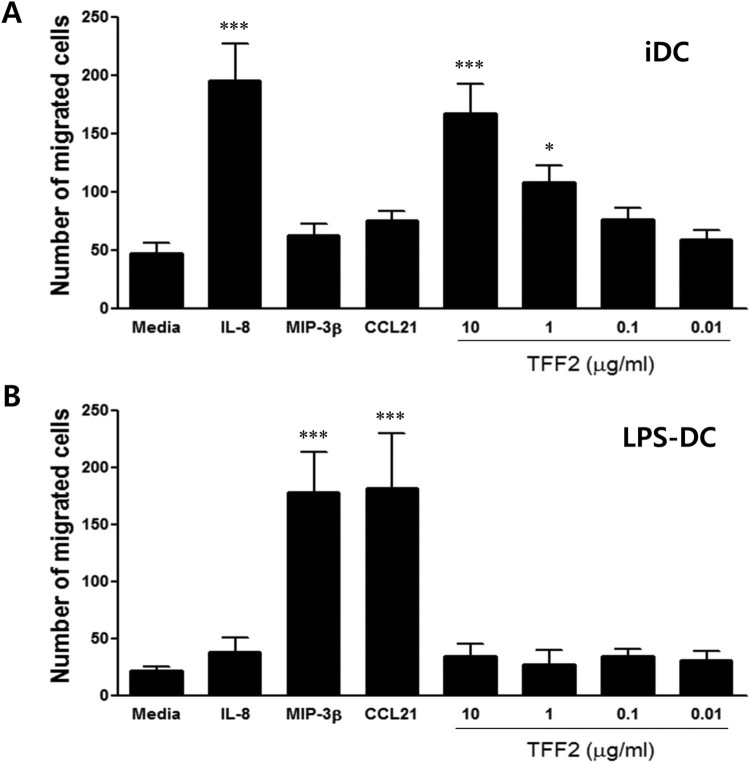
TFF2 induces the chemotaxis of iDCs, but not mDCs. Human monocyte-derived iDCs (A) or LPS-matured DCs (B) were analyzed for migration toward CXCL8 (IL-8, 50 ng/ml), CCL19 (MIP-3β, 10 ng/ml), and TFF2 (1μg/ml) in transwell assays. While iDCs migrate efficiently to IL-8 and TFF2, LPS-DCs were only attracted to MIP-3β. Results are shown as mean migrated cells ± SD of triplicate determinations and are representative of three independent experiments. *P < 0.05 and ***P < 0.001 versus that of media.

Acquisition of migratory capacity to the secondary lymph node via chemokine-chemokine receptor interaction is a hallmark of DC maturation and is essential for induction of T cell-dependent immune responses against pathogens. We examined the *in vitro* migratory capacity of immature and mature DCs in response to IL-8 (CXCL8) and MIP-3β (CCL19), which are chemotactic to immature DCs and LPS-DC, respectively. As shown in Figure 5, immature DCs exhibited a strong migratory capacity to IL-8, whereas LPS-mature DCs showed enhanced migration to MIP-3β. While TFF2 induced the migration of immature DCs in a dose-dependent manner, LPS-stimulated DCs exhibited poor migratory activity in response to TFF2, implying that TFF2 secreted by tumor cells is a chemoattractant for immature DCs, preventing their migration to lymph nodes from tumors.

## Discussion

Several reports have described tumor tissue as an immunocompromised environment, with circulating and tumor-infiltrating DCs being functionally defective in tumor patients (Orsini et al. 2003; Bang et al. 2006; Shurin et al. 2013). It is well established that tumor cells secrete a number of molecules that can negatively affect the function of immune cells. The majority of molecules that have been identified in this context thus far are chemokines and cytokines such as VEGF, IL-10, and TGF-β, which impair the function of effector T cells and DCs by altering the phenotype or by enhancing spontaneous apoptosis. Some of these findings have been associated with poor prognosis in patients. However, the release of other undefined soluble factors by tumor tissue has also been shown to be a relevant mechanism of immunosuppression *in vivo* as well as *in vitro*.

In order to identify genes associated with immune escape, we focused on genes coding for secreted proteins from two pancreatic cancer microarray datasets from the Gene Expression Omnibus database. The majority of the genes identified in the present study have previously been reported as upregulated in pancreatic cancer tissues. The majority of secretome genes are associated with extracellular matrix (ECM), cell communication, cytokine, and protease activity. The upregulated gene list in the present study may serve as potential markers of pancreatic adenocarcinoma. Among these genes are CKLF, DKK1, DKK3, EFNA4, IGFBP3, KLK6, KLK10, LIF, MDK, MSLN, SHISA5, SFN, and TAGLN2. Of 106 secretome genes identified, TFF2 was selected for subsequent immunological functional study.

Trefoil factors are widely distributed secreted proteins of mucin-producing cells. Of three trefoil factor (TFF) family proteins of human and other mammals, the gastric TFF1 (pS2), the spasmolytic polypeptide (hSP, TFF2) and intestinal TFF3 (hP1.b/hTIF), trefoil factor 2 (TFF2) is mainly synthesized and secreted by the gastrointestinal tract (primarily by the stomach). The abundant expression of TFF2 in a site-specific pattern in the normal physiologic state, as well as its ectopic expression in various ulcerative conditions suggests an important role in mucosal defense and repair. Azarschab et al. reported that aspirin upregulates TFF2 expression in human gastric cancer cell lines (Azarschab et al. 2001), and May et al. concluded that TFF2 expressed in normal and malignant breast epithelial cells stimulates the migration of breast cancer cells (May et al. 2004). Cook et al. showed that TFF2 and TFF3 are expressed by lymph nodes, spleen, and the gastrointestinal tract (Cook et al. 1999). They also showed that in the spleen, these genes can be upregulated by experimental inflammation and are able to stimulate monocyte migration. Together, these observations suggest a potential role for TFFs in the immunological responses as chemokines (Baus-Loncar et al. 2005) that may control the migration of immune cells between tissues (Heirani-Tabasi et al. 2017).

More recently, studies have revealed that TFF2 contributes to the protection of mucosa from infection by suppressing Th1 response or driving Th2 response (McBerry et al. 2012; Wills-Karp et al. 2012). A potential role of TFF2 in pancreatic cancer cell migration was demonstrated in pancreatic cancer cell lines (Guppy et al. 2012). Although CXCR4 was identified as a low-affinity signaling receptor for TFF2 (Dubeykovskaya et al. 2009), the expression of this chemokine receptor on DCs is not significantly modulated by maturation (Luft et al. 2002), implying that TFF2-induced migration of immature DCs is mediated by other signaling receptor(s) (Madsen et al. 2010). Yet, there is no conclusive report that TFF2 plays a role in dendritic cell function and pancreatic cancer immunity. The gene for the 25-amino-acid peptide neuromedin U (NMU) also exhibited 2.9 fold upregulation in the pancreatic cancer database, and previous studies (Johnson et al. 2004; Moriyama et al. 2006) imply that this protein plays a role in proinflammation, without affecting tumor cell growth.

Cancer specificity is one of the key requirements for diagnostic and/or therapeutic markers. We showed that the identified genes are from pancreatic cancer cell lines, and that they can modulate the function and/or maturation of DCs. We found that TFF2 are expressed in pancreatic cancer cell lines via RT–PCR as well as ELISA. These results are in sharp contrast with a recent study that examined the tumor suppressive role of TFF2 in human pancreatic ductal adenocarcinoma (PDAC) tissues and cell lines (Yamaguchi et al. 2016); the expression of TFF2 in PDAC was reduced compared to that of normal tissues, and transgene overexpression suppressed the proliferation of pancreatic cancer cell lines. The study revealed that TFF2 is expressed in pancreatic cancer cell lines while the expression appears to be epigenetically regulated; the TFF2 promoter was hypermethylation in TFF-2 low-expressing Panc-1 cells but not in TFF2 high-expressing AsPC-1 cells. Our data from 13 pancreatic cell lines reveal that a majority of them expressed *TFF2* transcript and secreted significant amounts of TFF2. Unfortunately, we were unable to find a correlation between TFF2 expression levels and cancer cell lines of different stages, as many of these tumor cell lines are of an uncertain origin and stage. Further studies are necessary to confirm the precise role of TFF2 in pancreatic cancer.

Although TFF2 marginally affected the phenotypic maturation of DCs by suppressing antigen presenting molecules (HLA-DR) and costimulatory molecules (CD40, CD80, and CD86), the allostimulatory capacity of LPS-induced mature DCs in the presence of TFF2 was unaffected. While TFF2 was a strong chemotactic factor for iDCs, it also effectively modulated the phagocytosis capacity of iDCs. Reduction in the endocytic activity of iDCs is evident in the presence of TFF2. Thus, TFF2 strongly attracts iDCs and inhibits the antigen uptake function of attracted DCs. Once DCs initiate the maturation process, mDCs become nonresponsive to the action of TFF2, possibly via downregulation of prospective receptor(s). The chemoattraction of immature DCs by tumor cells has clinical importance. The correlation of a high number of systemic tolerogenic/immature dendritic cells in the tumor microenvironement with poor prognosis was observed (Tjomsland et al. 2010), and the interaction between tolerogenic DCs and regulatory T cells has been reported in pancreatic cancer (Jang et al. 2017). These results strongly suggest that tumor-cell-derived TFF2 is a selective chemotactic factor for iDCs and may lead to deficiency of active DCs in the pancreatic cancer microenvironment. Upon sequestration of immature DCs within the tumor sites, the tumor may induce the maturation arrest of infiltrating DCs, or induce generation of tolerogenic DCs or myeloid-derived suppressor cells (MDSCs), leading to tumor tolerance and immune evasion.

In conclusion, we identified 106 highly upregulated genes for secreted proteins from a public database of pancreatic cancers and provide evidence that TFF2 modulates the function of human dendritic cells by acting as a chemokine for immature DCs and impairing their antigen uptake activity. This may be a general protective mechanism by TFF2 against the hyperactivation of DCs in pathogenic inflammatory conditions. In this regard, the identification of signal transduction events that participate in the differentiation inhibition and modulation of DC function by TFF2 would further contribute to the elucidation of the mechanisms underlying the complex anti-inflammatory effects of TFF2 and would allow the construction of a theoretical framework for its eventual therapeutic use. The impairments of DC function by tumor-derived cytokines/chemokines can deviate and compromise the possible T cell-mediated immune responses, leading to the immune escape of cancer. The results described here suggest that the presence of TFF2 in tumor tissue may participate in immunosuppression in synergy with other previously described immunosuppressive factors. Further studies are necessary to investigate the biological role of TFF2 in pancreatic cancers.
